# Lack of De‐Intensification Models for Female Head and Neck Cancer Patients With Favorable Prognosis

**DOI:** 10.1002/cam4.71734

**Published:** 2026-03-13

**Authors:** J. M. Vahl, J. Hahn, A. von Witzleben, S. M. Schroeder, C. Idel, B. Wollenberg, T. K. Hoffmann, S. Laban

**Affiliations:** ^1^ Department of Otorhinolaryngology and Head & Neck Surgery University Medical Center Ulm Ulm Germany; ^2^ Department of Otorhinolaryngology and Head & Neck Surgery University Medical Center Schleswig‐Holstein Lübeck Germany; ^3^ Department of Otorhinolaryngology and Head & Neck Surgery University Medical Center of the Technical University Munich Munich Germany

**Keywords:** alcohol, head and neck tumor, human papillomavirus, sex, smoking

## Abstract

**Purpose:**

The prognostic relevance of gender in head and neck cancer is often neglected. In contrast, the more favorable prognosis of human papillomavirus (HPV)‐associated oropharyngeal squamous cell carcinoma (OPSCC) is well recognized, and de‐escalation strategies are increasingly being tested in clinical trials. In this study, we focused on the prognostic significance of gender to clarify whether it should be given more attention.

**Patients and Methods:**

We requested data on patients with newly diagnosed head and neck cancer between 2002 and 2017 from the German Center of Cancer Registry (*n* = 212,920). First, we focused on sex‐specific survival according to primary tumor site in a nationwide context. Second, we examined a local dataset of 462 OPSCC patients, diagnosed at the University Hospitals of Ulm and Lübeck between 2005 and 2018 and followed up until 2024. Here we compared the prognostic value of sex with HPV status, alcohol consumption, and smoking habits (these parameters were not available at national level).

**Results:**

In the nationwide analysis, women showed better survival in all head and neck primary tumor sites studied. The mean survival difference was greatest in patients with OPSCC (1.3 years; *p* < 0.0001), followed by patients with nasopharyngeal carcinoma (1 year; *p* < 0.0001). In the subgroup of patients younger than the median age (62.8 years), women lived on mean 1.2 times longer than men (*p* < 0.0001). Besides, in the local dataset, men with HPV‐positive OPSCC did not have a better prognosis than women, even when HPV‐negative (*p* = 0.426). However, the prognostic effect of female sex may be attenuated in patients with high tobacco use; in this scenario, no sex‐related difference in survival was found (*p* = 0.56).

**Conclusion:**

Sex‐specific differences in survival, especially in OPSCC and nasopharyngeal cancers, suggest the implementation of gender‐sensitive oncology regarding prevention, treatment, and aftercare.

## 1 Introduction

We recently performed a nationwide analysis of 212,920 head and neck cancer patients in Germany between 2002 and 2017 [1, 2]. Hereby we examined tumor incidence, stage, gender distribution, travel distance, age‐, site‐, and diagnosis‐time‐specific survival. In particular, we confirmed gender as an independent prognostic factor and showed that the median overall survival of women exceeds that of men by an incredible 2 years [1]. This was a key finding which we would like to shed more light on in this work.

Although gender‐specific oncology is an emerging field of medicine involving pharmacologists and physicians, it has received little attention in treatment guidelines or clinical trials. However, gender differences in cancer development (including non‐sex‐specific cancers), treatment response, and side effects have been studied. Men are more likely to be affected and have a poorer prognosis, while women experience more side effects from cancer treatments [3]. Bladder tumors are an unclear exception to the rule of non‐sex‐specific cancers. Although women are less likely to be affected overall, the bladder tumors are more often detected at an advanced stage with a more aggressive course [4]. With regard to adverse events during chemotherapy, particularly hematologic toxicity, women have a 1.5‐ to 2‐fold higher risk of developing these [5]. They have higher blood drug concentrations and longer elimination times of cytotoxic agents than men [5]. It should be noted that drug approval studies have often been conducted in predominantly male populations [6]. The lack of focus in this important medical area has prompted, for example, the European Society for Medical Oncology to establish its own “Gender Medicine Task Force” [3]. Of course, gender in this context does not only mean physiological sex [7]. Nonetheless, it may be surprising that biological factors such as genetics and hormonal differences account for only about 20% of the overall sex difference in survival. Non‐biological factors such as lifestyle (substance use, diet, occupational health risks and risk behaviors) and social status (education, occupation, income and wealth) account for the remaining 80% [8]. This became particularly clear from a comparison of the life expectancy of nuns and monks (“Cloister‐Study” of the European Research Council), who do not differ from each other in their living conditions and occupational risks. While nuns have the same life expectancy as women in the general population, monks live up to 5 years longer than men in the general population [9]. This broad overview of the multifaceted nature of gender and its impact on cancer development, treatment tolerance and success already suggests that a one‐dimensional therapeutic approach for men and women needs to be reconsidered.

If, on the flipside, we are not talking about the complex construct of gender, but about a scientifically well‐founded laboratory parameter, namely HPV status, it seems to be more accepted among scientists that it is important for an accurate diagnosis (tumor/nodal/metastasis [TNM] status) and prognosis of cancer patients—more specifically for OPSCC. However, a closer look reveals that positive HPV status, like gender, is associated with biological and especially several other sociodemographic factors. Biologically, the integration of the viral oncoproteins E6 and E7 clearly plays a decisive role in HPV‐driven tumors [10]. Socio‐analytically, patients with HPV‐positive tumors are significantly more likely to be non‐smokers and younger than patients with HPV‐negative tumors [11]. Furthermore, HPV‐positive OPSCC predominantly affects Caucasian, middle‐class men [11]. At the timepoint of diagnosis, patients with HPV‐positive OPSCC present comparatively frequently with low T and high N status [11]. The significant mortality related discrepancy between HPV‐positive and ‐negative OPSCC has even made the American Joint Committee of Cancer reclassifying the TNM/UICC 8th edition using the surrogate marker p16 [12]. It might be noted hereby that adding HPV DNA to p16 immunohistochemistry is more cost‐intensive, but also more specific and prognostically relevant (particularly advantageous if double positive) [13, 14]. Expressing it with numbers, the average 5‐year survival rate of HPV‐positive OPSCC patients is about reasonable 80%–85% [15]. Post‐therapeutically, patients with HPV‐positive OPSCC thus have an approximately 30% better 5‐year survival across all treatment modalities compared the HPV‐negative ones [16]. Somehow, despite the downgrading of p16‐positive tumors, treatment has remained nearly unchanged [12]. What is quite new, however, is the recommendation of vaccination, routinely for boys and girls from the age of 9 years and a booster vaccination up to the age of 26 years for females and 21 years for males [15] (therapeutic vaccinations are currently only used in the context of trials, for example, as an adjunct to checkpoint blockade in the distant metastatic tumor stage [17]).

In this respect, we would like to proceed as follows: first, we will focus on sex‐specific survival by primary tumor site in a national context; and second, we will compare the prognostic value of gender with the well‐established prognostic value of human papillomavirus (HPV) status by additionally examining a local dataset of oropharyngeal squamous cell carcinoma (OPSCC) patients with available HPV status (not available in the national dataset). In this local subpopulation of OPSCC patients, we will also examine the prognostic effect of nicotine and alcohol consumption in relation to gender.

## 2 Patients

### 2.1 Nationwide Head and Neck Cancer Study in Germany

Data collection from the German Center for Cancer Registry Data (ZFKD). We requested data on patients with newly diagnosed head and neck cancer between 2002 and 2017 and received data on 212,920 head and neck cancer patients, including sex, age, TNM status, rounded date of diagnosis, and date of mortality follow‐up (East Germany: December 2015, West Germany: December 2017) with the endpoint vital status [1, 2]. The calculation of the UICC tumor stage was based on the 8th version, which was simplified to four stages without subgroups and included all oropharyngeal cancers according to the p16‐negative classification (missing HPV status in the registry data). Because of the lack of information on HPV status or substance use, we needed a second local data set to address our full research question, which is described below.

#### 2.1.1 Data Exclusion

Importantly, the state of Baden‐Württemberg only started reporting data to the cancer registry in 2009, after the reporting of cancer registry data became mandatory. In addition, there seemed to be a delay in reporting in 2016 and 2017, especially in eastern Germany. For survival analyses, head and neck cancer patients with missing data on survival time and status were excluded. Patients marked as deceased but without a date of death and those alive but without a date of last follow‐up or with only a death certificate were also excluded for survival analyses. Thus, the cohort available for survival analysis was reduced to 193,877 patients.

### 2.2 Regional Oropharyngeal Cancer Study in Ulm and Lübeck (Germany)

#### 2.2.1 Data Collection University Hospitals of Ulm and Lübeck

A retrospective cohort study, approved by the Ethics Committee of Ulm University (#176/19), was conducted with a total of 462 patients with OPSCC. Patients with the first diagnosis of OPSCC between 2005 and 2018 were included. Survival status was followed until December 2024. Time of diagnosis, age, gender, substance use, HPV status, TNM status, tumor stage (version eight), recurrence, and survival data were collected. In this study, positive HPV status is not only synonymous with p16‐positive but also with HPV DNA‐positive. The TNM status was simplified into four T subgroups and four N subgroups.

## 3 Methods

### 3.1 Data Analysis

The German Center for Cancer Registry provided summarized data in an Excel file. Data were imported into IBM SPSS Statistics 29 and GraphPad Prism 10 for statistical testing. Figures were prepared in GraphPad Prism and Microsoft Power Point 2019, and tables were prepared in Microsoft Excel 2019. Local data were treated in the same way, except that the values of interest were entered directly into IBM SPSS Statistics 29.

### 3.2 Statistical Analysis

Survival data were calculated using the Kaplan–Meier estimator. Differences in survival data were tested with (pairwise) log‐rank tests. Multivariate analysis was performed by Cox regression after model adjustment by omnibus tests, which provided hazard ratios (HR) with 95% confidence interval (CI) and level of significance (p). Moderation analysis within the Cox regression model was performed according to the Baron & Kenny method. Mann–Whitney *U* test was used when we wanted to test two independent samples for differences in their central tendency, but no normal distribution was given. To compare two nominally scaled variables based on the observed frequencies, we used the chi square test.

## 4 Results

A total of 212,920 head and neck cancer patients diagnosed between the years 2002 and 2017, mostly men (77%), were included in the analysis of German national cancer registry data. The UICC tumor stage distribution according to gender was similar among the different tumor primary sites. 69.8% of men and 65.5% of women were diagnosed with advanced cancer (UICC II, IV). For more descriptive demographic and clinical aspects, we may refer to our previous analyses including tables with sex and age distribution and residence [1, 2]. To further estimate the impact of sex on survival in the different primary tumor sites, we performed grouped survival analyses by primary site. In fact, we found that there was a significant difference for each head and neck tumor site studied.

### 4.1 Far Better Survival for Women With Head and Neck Cancer in Germany

**FIGURE 1:**
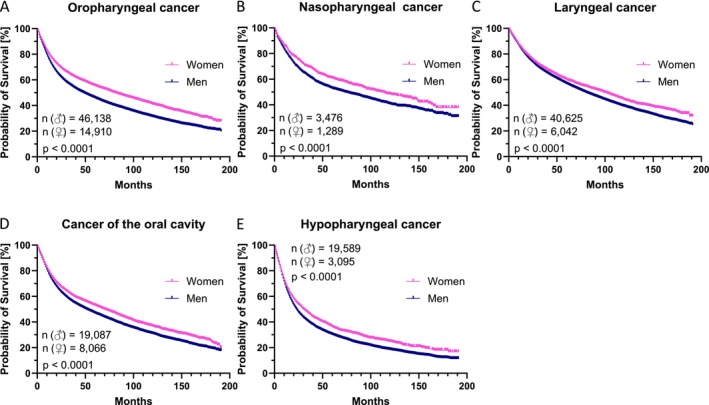
Overall survival of head and neck cancer patients in relation to gender in Germany. In the Kaplan–Meier curves (A)–(E) we show the overall survival [months] of 193,877 patients with oropharyngeal (A), nasopharyngeal (B), laryngeal (C), oral cavity (D), and hypopharyngeal cancer (E), diagnosed between 2002 and 2017 in Germany. Sample sizes are given in each illustration with “*n*.” Log rank test results are indicated with the significance level “*p*.”

The mean sex‐specific survival disparity was greatest in patients with OPSCC (Figure 1A). In 61,048 OPSCC patients, mean overall survival was 79 months (25,880 events; CI: 77.9–79.5) for men (*n* = 46,138) and 95 months (6896 events; CI: 93.4–96.5) for women (*n* = 14,910; *p* < 0.0001), which is an difference of 1.3 years. This discrepancy in survival is closely followed in patients with nasopharyngeal carcinoma (Figure 1B). In 4725 patients, mean overall survival was 95 months (1692 events; CI: 91.6–97.7) for men (*n* = 3476) and 107 months (526 events; CI: 102.0–112.3) for women (*n* = 1289; *p* < 0.0001), which is a difference of 1 year. The series further included 46,667 patients with laryngeal cancer (Figure 1C), 27,153 patients with oral cancer (Figure 1D), and finally 22,684 patients with hypopharyngeal cancer (Figure 1E). For the last three tumor types, mean survival in men (*n* = 40,625, 19,087, 19,589) was 95, 79, and 55 months (19,827, 10,949, 13,869 events; CI: 94.0–95.8, 77.3–79.7, 53.7–55.7). For women (*n* = 6042, 8066, 3095), mean survival was accordingly 103, 89, and 65 months (2581, 4055, 1977 events; CI: 101.0–105.7, 86.6–90.5, 62.4–68.2). All these differences in survival (8, 10, 10 months) are statistically significant (*p* < 0.0001).

We then sought to determine whether sex had a significant impact on survival in head and neck cancer patients independent of commonly known prognostic factors such as tumor stage and age.

### 4.2 Female Sex Is an Independent Prognostic Marker of Survival in Relation to Tumor Stage, and Age

**FIGURE 2:**
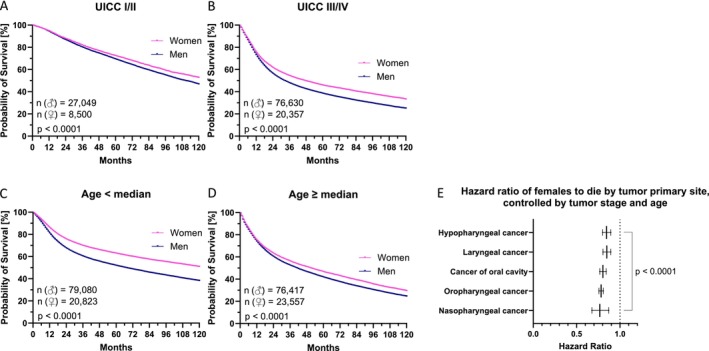
Sex‐specific survival in relation to tumor stage and age in Germany. In the Kaplan–Meier curves we show the Germany‐wide, sex‐specific overall survival [months] of head and neck cancer patients concerning low (A) and high (B) tumor stage as well as age below (C) and from the median on (D). Sample sizes are given in each illustration with “*n*.” Log rank test results are indicated with the significance level “*p*.” The forest plot (E) reflects the hazard ratios for female (vs. male) patients to die for each examined tumor primary site. These hazard ratios were controlled by age and tumor stage for each tumor primary site and are given with CI in the form of whiskers and significance level “*p*.”

Looking at the Kaplan–Meier curves of 112,995 patients with mixed head and neck cancer with low tumor stage (UICC I and II), we saw a clear difference (*p* < 0.0001) in survival according to sex (Figure 2A). Men (*n* = 27,049) lived a mean of 111 months (10,316 events; CI: 110.3–112.5), while women (*n* = 8500) lived a mean of 119 months (2695 events; CI: 116.7–120.8). The discrepancy (*p* < 0.0001) was also evident and even more obvious in the 70,165 patients with various high stage head and neck cancers (UICC III and IV). Here (Figure 2B), of course, overall survival decreased dramatically. Mean survival was 68 months (47,766 events; CI: 66.9–68.1) for men (*n* = 76,630) and 81 months (10,973 events; CI: 79.4–81.9) for women (*n* = 20,357). It should also be noted that the proportion of patients with less advanced tumors was not lower in women (26% vs. 29%). We also examined the value of sex in relation to median age (62.8 years) as a prognostic marker for survival in patients with various head and neck cancers. In 99,903 younger patients below the median age (Figure 2C), the mean survival of male (*n* = 79,080) patients was 90 months (41,036 events; CI: 89.7–91.0) and that of female (*n* = 20,823) patients was 111 months (8243 events; CI: 109.6–112.1). In the older group (Figure 2D), including patients aged at least 62.8 years, survival differed less pronounced (*p* < 0.0001); mean survival for men (*n* = 76,417) was 69 months (42,715 events; CI: 68.1–69.3) and for women (*n* = 23,557) 77 months (12,903 events; CI: 75.6–77.9). These latter results suggest an increased prognostic value of female gender in young patients (women live 1.23 times longer than men, *p* < 0.0001).

Finally, the independent hazard ratios for death using female sex as a predictor (Figure 2E), controlled for tumor stage (UICC I, II vs. III, IV) and age (below vs. at least median age) at diagnosis, were 0.77 (CI: 0.68–0.87) for nasopharyngeal cancer, 0.78 (CI: 0.76–0.81) for OPSCC, 0.80 (CI: 0.77–0.84) for cancer of the oral cavity, 0.85 (CI: 0.80–0.89) for laryngeal cancer, and 0.85 (CI: 0.80–0.89) for hypopharyngeal cancer.

We then wanted to further investigate the effect of sex on survival in relation to HPV status, smoking, and alcohol consumption. As HPV status and information on smoking and drinking habits were not available in the nationwide dataset, we used a local dataset from the University Hospitals of Ulm and Lübeck, including only OPSCC patients, collected between 2005 and 2018 (*n* = 462) and followed up until 2024, to complement our analysis. Their median age was 61.25 years (SD = 9.73). A total of 159 were HPV‐positive and 238 were HPV‐negative (65 patients with missing data). Alcohol and tobacco use were recorded in 404 and 373 patients, respectively. The proportion of female patients was only 20%, which may limit the power to detect an effect of gender on survival due to the smaller absolute population size. Other characteristics such as the distribution of TNM and HPV status are shown in Table 1.

**TABLE 1:** Characterization of the local OPSCC cohort. The included OPSCC patients are listed in a table sorted by clinical, simplified TNM status version eight and sex. The total and relative number of participants of each group is given. Furthermore, groups are divided by the proportion of patients with a positive HPV status.

		Number and percentage		HPV+/HPV− and percentage of HPV+	
T	T1	75	16.20	26/39	5.63
	T2	176	38.01	70/84	15.15
	T3	102	22.03	35/57	7.58
	T4	75	16.20	21/40	4.54
	Tx	9	1.94	2/4	0.43
	Missing	26	5.62	83	17.97
N	N0	88	19.01	22/61	4.76
	N+	308	66.52	132/138	28.56
	Missing	67	14.47	108	23.38
M	M0	429	92.66	153/219	33.12
	M1	8	1.73	3/5	0.65
	Missing	26	5.62	82	17.75
Sex	Men	342	73.87	124/177	26.84
	Women	87	18.79	32/48	6.93
	Missing	34	7.34	81	17.53
Age (years)	< 50	49	10.61	15/25	37.50
	50–69	302	65.37	124/142	47.37
	≥ 70	89	19.26	45/33	57.69
	Missing	22	4.76	78	16.88

### 4.3 Men With HPV‐Positive OPSCC Do Not Have a Better Prognosis Than Women

**FIGURE 3:**
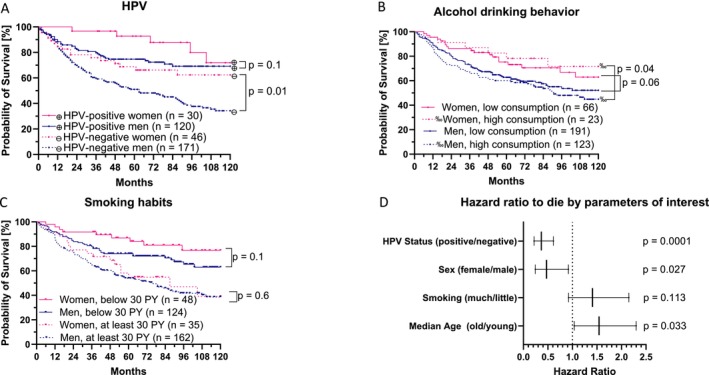
Sex‐specific 10‐year survival of local OPSCC patients by HPV status, alcohol drinking and smoking habits. In the first Kaplan–Meier curve (A) 10‐year survival of 462 OPSCC patients from Ulm and Lübeck is shown, considering the combined covariates HPV status and gender. The second and third Kaplan–Meier curves illustrate their sex‐specific survival concerning alcohol drinking behavior (B) and smoking habits (C). Sample sizes are given in each illustration with “*n*.” Log rank test results are indicated with the significance level “*p*.” The forest plot (D) displays the hazard ratios of OPSCC patients to die for HPV status (positive vs. negative), sex (male vs. female), smoking habits (< 30 pack years vs. at least 30 pack years), and median age (at least vs. below 62.8 years). These hazard ratios are given with CI in form of whiskers and significance levels “*p*.”

When we looked at the 10‐year survival of OPSCC patients (Figure 3A) compared by HPV status (positive vs. negative) and gender (male vs. female), the expected gap between HPV‐positive and ‐negative patients in general (*p* < 0.0001) as well as between men and women (*p* = 0.01) was detected, validating the cohort. The mean overall survival for men (*n* = 342) was 77 months (CI: 72–83 months) and for women (*n* = 87) 93 months (CI: 84–101). The mean survival for HPV‐negative patients was 70 months (CI: 64–77) and 95 months (CI: 88–102) for HPV‐positive patients.

When further subdivided, the mean survival of 171 HPV‐negative men was 66 months (101 events; CI: 59–73) and 46 HPV‐negative women lived for a mean of 86 months (16 events; CI: 72–100; *p* = 0.01). The mean survival of 120 HPV‐positive men was 92 months (34 events; CI: 84–100), while that of 30 HPV‐positive women was 108 months (5 events; CI: 99–118; *p* = 0.1). Therefore, survival was not significantly different by gender in HPV+ OPSCC patients. The slightly different overall prognosis in this local data set compared to the nationwide OPSCC data set may be due to the smaller population size or a less reliable recording of deaths (regular tumor follow‐up ends after 5 years, so that many patients automatically withdraw from the study observation). However, it is interesting to note that the survival of HPV‐positive men is comparable as that of HPV‐negative women (*p* = 0.426).

### 4.4 In Patients With High Tobacco Consumption the Prognostically Favorable Effect of Female Sex May Weaken

Then we continued the analysis with respect to smoking and alcohol consumption. The median amount of accumulated pack years was 30 for men and 20 for women (*p* = 0.011). Furthermore, 62% of men and 25% of women had a history of high alcohol drinking behavior (*p* = 0.34). We found no difference in survival with respect to total alcohol consumption (*p* = 0.31). Patients (*n* = 257) who never or only occasionally drank alcohol lived for a mean of 83.8 months (CI: 78.1–89.5), whereas patients who were daily or former heavy drinkers (*n* = 146) lived for a mean of 78.9 months (CI: 71.3–86.5). When divided by gender (Figure 3B), a significant difference in survival was observed for patients with high alcohol consumption only. The mean survival of patients in the low alcohol consumption cohort was 80.2 months (78 events; CI: 73.4–87.0) for men and 93.7 months (19 events; CI: 83.6–103.8) for women (*p* = 0.058). In the heavy drinking cohort, it was 75.2 months (63 events; CI: 66.8–83.6) for men and 98.8 months (6 events; CI: 83.4–114.1) for women (*p* = 0.038).

In contrast, when OPSCC patients were divided by the median number of overall pack‐years (30 pack‐years), a significant difference in survival was seen. Patients with < 30 pack‐years (*n* = 173) had a mean survival of 94.5 months (CI: 88.2–100.7), whereas patients with ≥ 30 pack‐years (*n* = 197) had a mean survival of only 71.2 months (CI: 64.5–77.8) (*p* < 0.0001). When we divided the groups according to smoking habits and gender (Figure 3C), we observed a mean survival of 90.9 months (39 events; CI: 83.2–98.7) for men with less than 30 pack‐years and 103.1 months (9 events; CI: 93.0–113.3) for the corresponding women (*p* = 0.01). In the group of patients with at least 30 pack years, mean survival was 70.1 months (87 events; CI: 62.7–77.4) and 76.2 months (17 events; CI: 61.0–91.4), respectively (*p* = 0.56). In conclusion, at this stage, there is no clear influence of gender on overall survival, at least in the group of patients with high tobacco consumption.

Finally, we examined hazard ratios for death comparing sex, HPV status, and smoking habits, controlled for median age (Figure 3D) in a multivariable analysis. Alcohol consumption was not included because there was no difference in the survival analysis in the univariate assessment. The hazard ratios were 0.37 for HPV‐positivity (CI: 0.22–0.61), 0.47 for being female (CI: 0.24–0.92), 1.41 for having at least 30 pack‐years (CI: 0.92–2.15), and 1.54 (CI: 1.04–2.30) for being older than the median age. The significance levels (*p*) were 0.0001, 0.027, 0.113, and 0.033, respectively, identifying smoking habit as a non‐independent predictor. However, an imminent moderation of smoking habit by gender could not be assumed in this cohort according to Cox regression modified by Baron and Kenny (*p* = 0.47).

## 5 Discussion

To the best of our knowledge, we are the first to report a nationwide gender gap in survival among patients with head and neck cancer in Germany by primary tumor site and under consideration of HPV status. The discrepancy in mean survival was largest in patients with OPSCC and nasopharyngeal cancer. Furthermore, we found that in our local OPSCC cohort, HPV‐positive men did not have a significantly better survival rate than HPV‐negative women, highlighting the prognostic value of gender, also in relation to HPV. However, smoking and alcohol consumption did not appear to have a significant effect in the multivariate analysis between sex, age, and HPV status. The last variable is probably the one that gets the most attention at the moment, although it only really plays a role in OPSCC. So, is there too little focus on sex in this context?

Of course, we now know that the mortality rate of HPV‐positive OPSCC patients is lower than that of HPV‐negative patients and that HPV‐positive OPSCC shows a significantly better response than HPV‐negative OPSCC, especially with regard to radio(chemo)therapy [18]. A classical, potentially curative treatment regimen consists of cisplatin (100 mg/m^2^ KOF) administered every 3 weeks and a radiation dose of 70 Gy over 6 weeks [19]. Here, it should be emphasized that the superior overall survival of HPV‐positive OPSCC is particularly evident in the low‐risk group (TNM 7: T1‐T3 N0‐N2, non‐smokers), as it was demonstrated in the retrospectively performed subgroup analysis of the Radiation Therapy Oncology Group (RTOG) 0129 study [19]. They showed a 2‐year survival rate of 87.5% vs. 67.2% for HPV‐positive or ‐negative OPSCC [20].

This observation was being followed by de‐escalation trials to investigate whether it is possible to reduce the morbidity of affected patients by reducing the extent of treatment while maintaining the better prognosis for HPV‐positive OPSCC.

Various approaches to reduce toxicity and morbidity during primary radiation therapy, especially in the low‐risk setting, included reducing the radiation dose in the NRG Oncology HN002 to 60 Gy and attempting to omit cisplatin (inclusion criteria: T1‐T2 N1‐N2b M0 or T3 N0‐N2b M0, up to 10 pack‐years [21]) or replacing cisplatin with cetuximab in the De‐ESCALaTE trial (inclusion criteria: < cN2b, up to 10 pack‐years [22]) and the RTOG 1016 trial (inclusion criteria: T1‐T2, N2a‐N3 or T3‐T4, N0‐N3 M0 [23]). While the replacement of cisplatin with cetuximab was not successful (De‐ESCALaTE, RTOG 1016), reducing the radiation dose while maintaining cisplatin was found to be a possible alternative therapy for HPV‐positive, low risk OPSCC patients (NRG Oncology HN002).

Another important approach is therapy de‐escalation in the context of adjuvant treatments. The risk‐adapted reduction of adjuvant radiotherapy or the omission of concomitant chemotherapy despite extracapsular spread (ECS+) after previous transoral tumor resection is currently tested in the PATHOS trial (inclusion criteria: T1‐T3, N0‐N2b [24]). This study could be quite promising, as it was previously shown in the ECOG 3311 study (inclusion criteria: T1‐T3 N0‐N2b M0 [25]) that adjuvant de‐intensification might be possible for patients with low‐risk OPSCC concerning radiation dose and chemotherapy. In addition to an adjuvant dose reduction, the AVOID study (inclusion criteria: T1‐T2 N1‐3 M0 [26]) also drew attention to the possibility of reducing the radiation field in HPV‐positive OPSCC patients.

If this much energy can be put into de‐escalating the treatment of HPV‐positive OPSCC, should we not also ask whether it is time to start de‐escalation trials in women, where female sex is a possibly no less relevant marker for even more tumor entities, and whether female sex may be included in the prognostically relevant TNM or UICC classification in a similar way to HPV status?

As mentioned in the introduction, both gender and HPV status are multifaceted constructs with a biological and social backbone. A de‐escalation study, for example, of radio‐chemotherapy in women, could also be called a sex‐specific dose‐finding study. Because clinical trials have often been conducted in populations that are predominantly male or rather for the stereotypical 70‐kg man, different body compositions have been neglected [6, 27]. Here, in the era of precision medicine, fat‐free muscle mass is suggested by some authors to become a novel parameter for drug dosing in oncology [5].

In tandem with the realization of the gender gap and the awareness of previous studies, it is also necessary to identify possible underlying reasons to be able to offer optimally individualized oncological treatment. In this context, biological reasons for the gender gap in cancer survival are the protective effects of X‐chromosome genes in females and androgen‐induced immunosuppression [4, 28]. More specifically, an X chromosome can be inactivated in the event of a deleterious mutation in a cell, a mechanism that only females can utilize [28]. Furthermore, androgens make innate immune cells such as neutrophils, monocytes, and macrophages increase the liberation of the anti‐inflammatory Interleukin‐10 [29]. Androgens also reduce the number of T cells and limit adaptive T cell responses by decreasing the production of inflammatory interleukin‐5 and interferon‐gamma and increasing the production of interleukin‐10 [29]. In contrast, regardless of tumor diseases, estrogen induces the expression of longevity‐associated genes, including superoxide dismutase and glutathione peroxidase, making especially mitochondria from females produce fewer reactive oxygen species [30]. Epigenetic sex differences in DNA methylation patterns, also regarding tumor suppressor genes, appear to play a role too [31]. In a mouse model, it has even been shown that HPV‐positive head and neck tumors in male mice also grow faster and respond less well to radio‐chemotherapy than in female mice, although tumor‐free female mice were more fatigued by cisplatin therapy than male mice [32]. This experiment underlines the tumor‐relevant, biological difference between men and women, regardless of their social behavior. This may also be one of the reasons why the gender gap was greater in younger patients in our analysis, or rather because the proportion of premenopausal women was higher than in the older group. Nevertheless, in addition to these impressive biological differences, non‐biological factors account for the majority of the discrepancy in survival between men and women in Germany [8]. An especially important aspect seems to be the unequal smoking behavior [8], an effect of which we could also observe in absolute and relative metrics in the survival analysis of our OPSCC patients. However, in this respect we were unable to determine any significant moderation of smoking behavior by gender. It is also interesting to note in addition that smoking not only promotes the development of cancer but also has a significant influence on tumor biology. Smoking increases proliferation, migration, invasion, angiogenesis and the activation of anti‐apoptotic signaling pathways in tumor cells [33]. It also has an immunosuppressive effect on the tumor microenvironment in head and neck tumors, for example, by decreasing the invasion of cytolytic tumor cells [34]. Alcohol toxicity is otherwise caused by ethanol and its metabolites directly at the DNA level and additionally, for example, by influencing the microflora, producing oxidative stress [35]. Therefore, supplementary to the development of a gender‐specific therapy, it would absolutely make sense to implement a gender‐specific prevention program. Such program would particularly have to include an anti‐smoking campaign for men.

These kinds of initiatives should be considered not only in Germany, but internationally. An analysis of the Surveillance, Epidemiology, and End Results database (USA) also pointed out that the incidence‐based mortality rates for pharyngeal cancers differ significantly according to gender (1:3), to the disadvantage of men [36]. Similarly, data from China on nasopharyngeal carcinoma show a gender‐specific survival advantage for women, specifically those individuals who are not postmenopausal [37].

As a subsidiary comment, we would like to add here that the broad field of potential gender‐related frictions affects not only patients but also scientists. Structural sexism, meaning the unequal distribution of resources and power, in oncology could be an obstacle to this type of women‐friendly research [38]. For example, in terms of the quantitative output of oncological publications, female authors are underrepresented and receive less recognition than men [39].

Oncologic de‐escalation trials exclusively for women are currently performed only in one sex‐specific tumor, early breast cancer [40]. Even in general, de‐escalation trials are not yet common in oncology. In addition to breast and head and neck cancer, such trials are conducted only in chronic myeloid leukemia, medulloblastoma, and prostate cancer [41]. An interesting complementary approach is not to de‐escalate the actual therapy, but rather to optimize the precision and early mitigation of side effects—technical advances in imaging techniques and radiotherapy machines could play an important role here [19]. In order to further assess whether de‐escalation studies could also be useful in the palliative setting, the next step could be a new retrieval of current cancer registry data to examine the effect of gender on progression‐free survival in head and neck cancer patients. Another interesting aspect would be whether the manner of tumor aftercare should also be arranged differently for men and women.

What must not be ignored in all de‐escalation studies is that head and neck cancer patients are quite willing to accept morbidity for the chance of a cure [42]. Therefore, proven treatment regimens should continue to be offered primarily at the present time and de‐escalation strategies should only be pursued after appropriate patient education in trials [43]. Good communication and trust in clinicians appear to be essential for patients who nevertheless accept the risk of a de‐escalation trial in the hope of reduced morbidity [41].

## 6 Conclusion

For head and neck cancer patients in Germany, there are occasionally large gender‐related differences in survival, especially for OPSCC and nasopharyngeal cancers. Therefore, gender‐specific oncological therapy and especially educational measures should be established in accordance with the philosophy of precision medicine. Presumably, women of younger age could benefit the most from therapy de‐intensification, while on the contrary, men may especially profit from preventive care expansion. Attempting to further characterize the biological factors that contribute to the gender gap could, of course, benefit both men and women in the development of new therapies.

## Author Contributions

Conceptualization: J.M. Vahl and S. Laban. Methodology: J.M. Vahl, S. Laban. Software: NA. Validation: J.M. Vahl, S. Laban. Formal analysis: J.M. Vahl, S. Laban. Investigation: J.M. Vahl. Resources: S. Laban, C. Idel, B. Wollenberg, T.K. Hoffmann. Data Curation: J.M. Vahl, S. Laban. Writing – original draft: J.M. Vahl, S. Laban. Writing – review and editing: J.M. Vahl, S. Laban, T.K. Hoffmann, J. Hahn, A. von Witzleben, S.M. Schroeder, B. Wollenberg, C. Idel. Visualization: J.M. Vahl. Supervision: S. Laban, T.K. Hoffmann. Project administration: J.M. Vahl. Funding acquisition: NA. The work reported in the paper has been performed by the authors, unless clearly specified in the text.

## Funding

The authors have nothing to report.

## Ethics Statement

All procedures were performed in accordance with the ethical standards of the institutional research committee and with the Helsinki Declaration of 1964 and its subsequent amendments or comparable ethical standards. The analysis of the local dataset was performed after approval by the Ethics Committee of Ulm University (#176/19). Moreover, in agreement with our ethics committee at the University of Ulm, no separate ethics review was required for the national data set because data were externally anonymized prior to analysis.

## Consent

Epidemiologic cancer registration in Germany is regulated by state laws, and the data received have been anonymized. The Federal Cancer Registry Data Act of 2009 defines the tasks of the Center for Cancer Registry Data at the Robert Koch Institute as the national evaluation center. This means that all physicians and dentists in the country are obliged to report cancer cases in which they are involved in the diagnosis, treatment, or follow‐up to the national cancer registry. Patient consent is not required.

## Conflicts of Interest

Simon Laban: Advisory Boards: Merck Sharp & Dohme (M.S.D.), Bristol Myers, Squibb (B.M.S.), Astra Zeneca (A.Z.). Honoraria: M.S.D., B.M.S., A.Z., Merck Serono. Thomas K. Hoffmann: Advisory Boards: M.S.D., B.M.S., Sanofi. Honoraria: M.S.D., B.M.S., Merck Serono. All other authors declare no conflicts of interest.

## Data Availability

The original data sets cannot be shared. The data on head and neck cancer in Germany have been requested from the German Cancer Registry in a multi‐step process for a specific purpose and a specific group of persons only and may not be shared. The data set on OPSCC patients is sensitive with regard to the protection of personal data and can also not be shared.
